# Hyaluronan Benzyl Ester as a Scaffold for Tissue Engineering

**DOI:** 10.3390/ijms10072972

**Published:** 2009-07-03

**Authors:** Vincenzo Vindigni, Roberta Cortivo, Laura Iacobellis, Giovanni Abatangelo, Barbara Zavan

**Affiliations:** 1 Unit of Plastic and Reconstructive Surgery, University of Padova, Via Giustiniani 2, 35100 Padova, Italy; E-Mail: vincenzo.vindigni@unipd.it (V.V.); 2 Dept of Histology, Microbiology and Biomedical Technologies, University of Padova Viale G. Colombo 3, 35131 Padova, Italy; E-Mails: roberta.cortivo@unipd.it (R.C.); giovanni.abatangelo@unipd.it (G.A.)

**Keywords:** HYAFF, hyaluronan, tissue engineering

## Abstract

Tissue engineering is a multidisciplinary field focused on *in vitro* reconstruction of mammalian tissues. In order to allow a similar three-dimensional organization of *in vitro* cultured cells, biocompatible scaffolds are needed. This need has provided immense momentum for research on “smart scaffolds” for use in cell culture. One of the most promising materials for tissue engineering and regenerative medicine is a hyaluronan derivative: a benzyl ester of hyaluronan (HYAFF^®^). HYAFF^®^ can be processed to obtain several types of devices such as tubes, membranes, non-woven fabrics, gauzes, and sponges. All these scaffolds are highly biocompatible. In the human body they do not elicit any adverse reactions and are resorbed by the host tissues. Human hepatocytes, dermal fibroblasts and keratinocytes, chondrocytes, Schwann cells, bone marrow derived mesenchymal stem cells and adipose tissue derived mesenchymal stem cells have been successfully cultured in these meshes. The same scaffolds, in tube meshes, has been applied for vascular tissue engineering that has emerged as a promising technology for the design of an ideal, responsive, living conduit with properties similar to that of native tissue.

## 1. Introduction

The major goal of tissue engineering is to replace, repair or enhance the biological function of damaged tissue or organs. The principal obstacle to clinical transplantation today is the shortage of donor organs. For this reason, tissue engineering and regenerative medicine have become promising and important fields of research, which may offer new sources of tissues and organs for transplantation. Repair of skin, bone, liver and blood vessels is likely to become of clinical interest when three dimensional cell and tissue reconstructive procedures are correctly matched with the appropriate supporting biomimetic materials.

Recent developments in the multidisciplinary field of tissue engineering have yielded many novel tissue replacements and implementation strategies. Scientific advances in biomaterials, stem cell isolation, growth and differentiation factors and biomimetic environments have created unique opportunities to fabricate tissues in the laboratory. One of the major challenges now facing tissue engineering is to optimize three dimensional (3D) functional recovery, a factor dependent on 3D structural complexity, as well as the biomechanical and functional stability of the laboratory-grown tissues destined for transplantation.

In the complex scenario of biopolymers useful on Tissue Engineering field hyaluronic acid holds an important position. Hyaluronic acid (HA) is a naturally occurring non sulphatated glycosaminoglycan consisting of a linear sequence of d-glucuronic and *N*-acetyl-d-glucosamine. It is present in connective tissue, in the synovial fluid of articular joints and in the vitreous humor of the eye. HA is important in many biological processes such as tissue hydration, cell differentiation, cell behavior and tissue repair. In recent years a novel hyaluronan polymer based scaffold has shown surprising properties in the field of tissue engineering [1,2]. This semysinthetic insoluble polymer obtained by the hyaluronic acid esterification with benzyl alcohol (HYAFF^®^ Fidia Advanced Biololymers - F.A.B Abano Terme, Italy) is biocompatible, completely biodegradable, soluble in DMSO, exhibits good stability to hydrolysis, forms contact angle measurements and presents a strong ability to interact with polar molecules. [3–5]

With the phase inversion technique, the esterification of all the carboxyl group with alcohols totally esterified hyaluronic acid derivatives (namely HYAFF11^®^) could be available in the form of films, non-woven fabrics, gauzes, sponges, tubes and microspheres. All these devices have been used in several medical applications for tissue repair, controlled drug release, nerve regeneration and as wound dressings or in microparticles easily enriching with active proteins (i.e. growth factors) for drug delivery application [6,7].

In the present review we report the most popular applications obtained with this biomaterial in tissue engineering, regenerative medicine and in clinical practice.

## 2. Skin

Successful treatment of burns and skin lesions requires the development of skin substitutes that can replace autografts. With the continued evolution of cell culture systems and the promising biological performance of three-dimensional support scaffolds, advancements have progressed rapidly in this field of tissue engineering. Initially, results obtained with autologous keratinocyte sheets, particularly in the treatment of burns, were encouraging. Kertinocytes obtained from a small patient biopsy are able to growth rapidly on a HYAFF11^®^ film, named Laserskin^®^, developing a keratinocyte culture/transfer device. Indeed, keratinocytes cold be transferred on the healing bed using this hyaluronic acid film. Laserskin^®^ is a sheet of maximally esterified (and therefore minimally-soluble) benzyl ester of hyaluronan (HYAFF11 material) in which micro perforations, with diameters of 40 mm and 500 mm, are made with a laser-drill [8–13]. Initially it was designed as a transfer mechanism for keratinocytes from tissue culture to wound bed. In fact cells applied to its surface *in vitro* were allowed to proliferate across it and migrate down the perforations. It could then be applied with the colonized surface uppermost, allowing the proliferating cells to populate the wound bed via the pores [14]. However, these constructs were too delicate and difficult to handle in the clinical setting due to its thinness. Researchers have therefore turned their attention to developing composite skin substitutes in which keratinocytes are cultured on an engineered dermal layer whose aim is to create an easy-to-handle and more resistant skin substitute. In keeping with the bilaminar concept, scaffolds created using modified hyaluronic acid have been employed for the co-culture of autologous fibroblasts and keratinocytes. In principle, the concept of *in vitro* organogenesis allowed the development of a two-layered living skin equivalent that consists of both dermal and epidermal cells resembling full thickness skin with a fibroblast layer and a well-developed multilayer epithelium [13]. Shortly after grafting, skin organotypic cultured grafts normalize their tissue architecture, basement membrane structure and barrier function [14,15]. Development of skin equivalents has progressed further by the addition of endothelial cells to co-culture systems, aiming to create a microcapillary network inside the dermal layer. Tonello and colleagues have obtained promising results by co-culturing three different human cell types - fibroblasts, keratinocytes and endothelial cells - obtained from full-thickness skin biopsies, onto a non-woven hyaluronic acid based scaffold (HYAFF11^®^) [15]. Initially, fibroblasts were seeded to allow for the production of the main extracellular matrix molecules necessary for proliferation and differentiation of keratinocytes and for endothelial cells proliferation and organization into capillary-like structures [15]. Extracellular matrix molecules secreted by fibroblasts (i.e. Collagen type I; collagen Type II; collagen type VI; fibronectin; laminin) provide the physiological environment for endothelial cell differentiation, keratinocytes have also been shown to play an important role by providing endothelial cells with the production of vascular endothelial growth factor (VEGF) [16]. Moreover, it is well known that in presence of hyaluronidase enzymes hyaluronic acid is catabolized by to either oligosaccharides or larger polymer and that these oligosaccharides stimulate cytokine secretion for endothelial cell proliferation; attachment and organization [17,18]. In turn, endothelial cells secrete an organized sub-endothelial matrix that is critical to the success of vascular grafts since the strength of the scaffold-matrix-cell interface determines the ability of the cells to resist blood flow. The presence of a microcapillary network should facilitate the take of a skin substitute, particularly for the treatment of full-thickness wounds. Furthermore, an endothelialized skin substitute should accelerate the revascularization process at the transplant site by inoculation of the capillary-like structures with the local wound vessels [15].

## 3. Cartilage

Injuries to articular cartilage are known to have limited self-healing capacity and if left untreated have the tendency to develop into early degenerative joint changes. Conventional surgical treatments for joint lesions are not fully satisfactory because they often result in a repair tissue that is fibrocartilaginous and exhibits poor mechanical properties [19]. Autologous chondrocyte implantation (ACI) was first introduced in Sweden in 1987 as an innovative therapeutic option alternative to traditional surgical methods for cartilage repair, such as bone marrow stimulation techniques and mosaicplasty. A large amount of evidence currently is available in the literature concerning the clinical results obtained with ACI indicating that the treatment is associated with substantial pain reduction and improved joint functionality, resulting in improved health and economic outcomes. These findings, together with the documented evidence of hyaline like cartilage formation at the graft site, in many cases support the hypothesis of this cell-based treatment being regenerative, not only reparative. However, despite the promising clinical results obtained so far, the use of ACI is associated with a number of limitations essentially correlated with the complexity and morbidity of the surgical procedure and the frequent occurrence of periosteal hypertrophy. To overcome these limitations, tissue engineering has emerged as an innovative field of research with the potential to recreate three-dimensional structures such as cartilage to be used as a replacement for damaged tissue and to regenerate a functional organ *in vivo*. Engineered cartilage, generated by chondrocytes expanded from a small tissue biopsy and cultured in a 3D environment (e.g., porous biodegradable scaffolds), has the potential to support the regeneration of large joint defects, allowing early postoperative rehabilitation and functionality. From a biologic point of view, the scaffold for cartilage engineering must promote chondrocytes attachment and in-vitro proliferation as well as favor the expression and maintenance of a cell differentiated phenotype. An appropriate polymer scaffold should allow for three-dimensional distribution of chondrocytes stimulating the synthesis of extracellular matrix molecules and preventing the loss of cell phenotype for a proper cartilage function once implanted [20]. Indeed, human articular chondrocytes derived from knee articular biopsies and cultured as a monolayer de-differentiate after a few days of cell expansion, but a three-dimensional environment promotes re-differentiation to the cartilage phenotype. RNA analysis and immunohistochemistry reveal that cells sustain the expression of cartilage genes and proteins, such as collagen type II, type IX, and type X, aggrecan and sox 9 [21,22]. In this contex, Hyalograft^®^ C Autograft, a commercial three-dimensional HYAFF11 scaffolds enriched with autologous chondrocytes, has reveled in clinical medium-term results on the treatment of 0.5 to 15 cm^2^ defects more than 90% of patients expressing an improvement in knee symptoms and functionality and a very limited number of complications. The Hyalograft® C Autograft was simply placed into the prepared lesion, where it was stable and without the need for any fixation method.

With regard to the direction of tissue regeneration overtime, histological observation of specimens demonstrated that, in most mixed tissue, regeneration occurred first in contact with the subchondral bone and then proceeded toward the outer region. Soon after grafting, chondrocytes are known to proliferate and to synthesize extracellular matrix cartilage with a poor molecular network rich in collagen type I leading to the formation of immature tissue that, in time, differentiate and become organized into hyaline tissue with cells in columns immersed in a specialized extracellular matrix rich in collagen type II. The Hyalograft C autograft is then defined as a tissue-engineered graft composed of autologous chondrocytes grown on a three-dimensional HYAFF11® scaffold. This innovative approach was first introduced into clinical practice in a number of European countries in 1999 for the treatment of full-thickness cartilage defects and has generated growing interest in the orthopaedic community with more than 4,800 patients treated so far. From several analyses of second-look and third-look cartilage biopsies from patients with cartilage lesions treated with Hyalograft® C Autograft, Brun *et al*. [23] stated that the plasticity of mature chondrocytes is sufficient to enable de-differentiated cells to revert to differentiation. For this reason, mature cells can be used in *in vitro* cartilage tissue reconstruction with impressive results in terms of grafting and clinical outcome [24,25].

More recent studies allowed favorably comparing this tissue-engineering based approach with the clinical outcome of ACI and so setting the superiority of cell-based treatments. The most relevant difference with the ACI technique remains the implantation method. The use of HYAFF11® matrix significantly facilitates the surgical procedure avoiding the use of a periosteal flap and allowing the use of minimally invasive techniques therefore reducing surgical time and morbidity. Overall, the results indicate that treatment with the Hyalograft C Autograft is associated with improvements in relief of symptoms, mobility, pain reduction and quality of repaired cartilage tissue in the majority of patients with a limited number of adverse events reported, hence representing a valid option for the treatment of cartilage lesions in the knee [26].

## 4. Adipose Tissue

The correction of soft-tissue defects presents a challenge in plastic and reconstructive surgery. The implantation of isolated and culture-expanded adipose precursor cells is a solution to this problem because these cells differentiate into adipocytes when implanted *in vivo*. While several experimental approaches using adipose derived stem cells (ADSC) with various biomaterials for adipose tissue engineering have been proposed, many of these biomaterials are currently not commercially available. Consequently, the identification of suitable scaffolds that are readily available for adipose tissue engineering is necessary in order to apply these technologies to realistic therapeutic strategies [27]. Candidates of such scaffolds include type I collagen sponge, non-woven polyglycolic acid (PGA) and hyaluronic acid gel. Hyaluronic acid gel has been applied as wrinkle filler in the field of cosmetic medicine [28]. Hyaluronic acid is one of the natural extracellular matrix compuoi and is reported to be a suitable scaffold for adipose tissue engineering. It was used for this purpose as a sponge or a gel. The *in vivo* efficacy of constructs in generating mature adipose tissue has been clearly correlated to the *in vitro* cell inoculation of the scaffolds and to the structure and type of biopolymer used [29]. It was demonstrated that open hyaluronan sponges with interconnecting pores of 50 to 340 μm had deeper penetration of adipocytes than either collagen sponges or non-woven hyaluronan meshes [30]. Even larger scaffold pores were shown to allow better cell penetration, although maturation of the progenitor cells was compromised [31]. In contrast to rigid biopolymer carriers, biological hydrogels such as fibrin and Matrigel (Becton Dickinson, Heidelberg, Germany) lack of structural rigidity and deform easily [32]. Matrigel has been demonstrated to effectively support angiogenesis and preadipocyte maturation both *in vitro* and *in vivo*. However, its use is restricted to experimental purposes because its origin is from a murine sarcoma. Therefore, it would be highly desirable if alternative gels with adipogenic characteristics were available for human clinical use. It would be advantageous to have an implantable biomaterial that would both support the growth of adipocytes and actively induce neovascularization of the graft. It is well known that hyaluronic acid and its derivatives are actively angiogenic [33]. Injectable hyaluronic acid-based gels mixed with undifferentiated pig preadipocytes were evaluated in an autologous pig model to analyze their suitability as filler for soft tissue reconstruction [33]. Two types of gels with varying degrees of amidation were tested [33]. Microscopical examination of explanted tissue samples revealed the formation of highly vascularized adipose. The small size of the adipocytes supported the belief that these fat cells were newly formed, differentiated (pre)adipocytes [33]. The adipose tissue was found around mature vessels in addition to newly formed small vascular structures. In summary, these findings demonstrated that hyaluronic acid gel is applicable for generating adipose tissue in gels, displaying adipogenic as well as angiogenic properties, and strongly indicate its use as a matrix material for use as an injectable strategy for soft tissue reconstruction [34].

## 5. Gland Tissue Reconstruction

Treatment of hepatic/pancreatic diseases has been greatly improved by the advent and evolution of liver/pancreas transplantation. Yet as demand for donor organs continues to increase beyond availability, the need for alternative therapies becomes evident. Several approaches, including extracorporeal devices, cell transplantation and tissue-engineered constructs, have been proposed as potential adjuncts or even replacements for transplantation. Tissue engineering of implantable cellular constructs is an emerging cellular therapy for hepatic disease. Hepatocytes and Langerhans islets known to be anchorage dependent, are immobilized on scaffolds, encapsulated in aggregates, or cultured ex vivo to form liver “organoids” that are then surgically transplanted [35]. In the first years of the XXIst century Catapano *et al*. reported a preliminary investigation on HYAFF11^®^ and hepatic cultures, [35] suggesting that polymers made of hyaluronan benzyl esters could be promising substrata for *in vitro* hepatocyte culture. Hepatocytes adherent to films of hyaluronan benzyl esters were as functional as cells adherent to collagen. Cells cultured in non-woven fabrics of the ethyl hyaluronan ester (HYAFF7^®^) exhibited an initial capacity to eliminate ammonia comparable to that of cells adherent to collagen but synthesizing urea at a significantly greater rate. In the long term, cells cultured on HYAFF7^®^ non-woven fabrics also retained much more of their initial metabolic activity. This latter feature makes these substrata particularly interesting for hepatocyte culture in bioreactors, where long-term maintenance of liver-specific function is an essential requirement for the success of the treatment.

Following on these consideration Zavan *et al*. [36,37] investigated whether the extracellular matrix produced by fibroblasts cultured on HYAFF11^®^ non-woven fabric constructs could influence hepatocyte morphology and metabolism. In addition, they investigated hepatocyte transplantation in the rat subdermis using the extracellular matrix enriched scaffold (EMES) as a carrier. Results indicated that hepatocytes cultured within an environment-enriched device such as these EMES scaffolds maintain their typical morphology during the entire culture period. With regard to metabolic activity, such as albumin secretion, urea synthesis and ammonia elimination, *in vitro* cultured hepatocytes exhibited a continuously decreasing metabolic capacity overtime. When cultures were implanted into the subdermal tissue of nude mice, hepatocytes were observed in the innermost regions of the scaffold, where they maintained their native hepatic cell morphology, pyramidal cell shape and large round nuclei, although their density tended to progressively decline, probably due to either apoptosis and/or an inability to self-renew.

As regards to beta cells of pancreatic origin, their cultures on HYAFF11^®^ sponges not only ensured a constant vitaly (MTT value did not show any increase in time) but also the cells are able to maintain their original phenotype (i.e. ditizon positive labeling: a specific vital dye used for pancreatic islet staining) [36,37].

## 6. Peripheral Nerve Regeneration

Peripheral nerve regeneration is a serious clinical problem. As early as 1880, experimental investigators and clinicians alike bridged the cut ends (stumps) of a peripheral nerve by inserting them in a tube (nerve chamber) fabricated from a large variety of materials. Still today, coaptation of the two nerve ends is commonly used to repair short nerve defects. When larger nerve gaps exist (20 mm or longer in humans), the current clinical gold standard for repairing larger nerve deficits involves using sensory nerve autografts. An analysis of clinical outcomes with autograft use suggests that a critical need for engineered alternatives exists. Autografts are plagued by issues such as a shortage of donor nerves, a mismatch of donor nerve size with the recipient site and occurrences of neuroma formation; even in the best-case scenarios complete recovery of function is rare. Driven by enormous clinical need, interest in peripheral nerve regeneration has become a prime focus of research and area of growth within the field of tissue engineering. Therefore, the need for synthetic alternatives to autografts is compelling and would be of great surgical benefit if they could match or exceed autograft performance. Experimental configurations that have maximized facilitation of peripheral nerve regeneration are those in which the tube wall comprised degradable polymers, including collagen and certain synthetic biodegradable polymers, and was cell-permeable rather than protein-permeable. Tube fillings that showed very high regenerative activity were suspensions of Schwann cells, a solution either of acidic or basic fibroblast growth factor, insoluble Extracellular matrix (ECM) substrates rather than solutions or gels, polyamide filaments oriented along the tube axis and highly porous, insoluble analogs of the ECM with specific structure and controlled degradation rate. These materials are attractive because their chemical and physical properties can be modified by physical coating, plasma treating and chemical bonding through adhesion mediators [38–40]. The majority of artificial polymers that have been studied belong to the polyester family, which includes polyglycolides and polylactides. Some disadvantages of these polymers in tissue engineering applications are their poor biocompatibility, release of acidic degradation products, poor processability and loss of mechanical properties very early during degradation. HYAFF11^®^ is known for its biodegradability and biocompatibility, as well as its capacity to promote adhesion and proliferation of different cell types. In light of these consideration Zavan *et al.* [41] tested the ability of this biomaterial to allow *in vitro* adhesion and proliferation of Schwann and endothelial cells obtained from the same peripheral nerve sample assuming that endothelial and peripheral nervous cells positively interact together to stimulate coordinated peripheral nerve and correlated microcapillary regeneration. The possibility of creating a microvascularized peripheral nerve substitute is very intriguing and opens up a completely new scenario for *in vivo* nerve regeneration.

Results confirmed that tube devices made of HYAFF11^®^ based scaffolds are a good substrate for both *in vitro* peripheral nerve cell culture and nerve explants. Cells derived from rat sciatic nerve can be plated on these scaffolds where they attach, proliferate and colonize all the device surfaces. The cell population obtained from nerve digestion is represented by Schwann cells, endothelial cells and fibroblast-like cells that can be co-cultivated to obtain a microvascularized nerve substitute.

This finding supports the evidence reported in recent literature of coordinated regeneration of the peripheral nerve and corresponding microcapillary network. Schwann cells secrete vasculogenic growth factor to stimulate contemporary microcapillary regeneration. The presence of a microcapillary network *in vitro* should improve the outcome of *in vivo* implantation, favoring prompt reconstitution of the vascular channels and the arrival of oxygen and nutrients to the cells inside the scaffold.

## 7. MSC Differentiation

Mesenchymal stem cells (MSCs) are pluripotent self-renewing cells capable of generating all major skeletal tissues, such as cartilage, bone, fat, tendon, muscle, when grown in appropriate *in vivo*/*in vitro* environments. MSCs have been successfully isolated from the bone marrow or from adipose tissue of various species including human, rat, mouse and rabbit. Aguiari *et al*. [42] reported the ability of adipose derived Mesenchymal stem cells to *in vitro* commit in adipose tissue, cartilage and bone on HYAFF11^®^ based scaffolds, whether Zavan *et al*. [43] reported that human and rat bone marrow derived MSC show the same behavior in terms of cell proliferation, differentiation and morphological organization in 3D scaffolds in presence of both cartilage or bone external stimulus. In light of their multilineage differentiating potential and their capacity to undergo extensive replication without losing this capacity, MSCs have an enormous potential in the fields of cell therapy and tissue engineering. Radice *et al*. [44] applied MSC for the repair of osteocohondral defect in rabbit knee. The starting idea of this experimental model was that MSC could be applied as valid alternative approach to differentiated chondrocytes for cartilage repair. It has been hypothesized that in adult life tissues, such as cartilage, cannot repair because of the lack of a sufficient quantity of mesenchymal precursors able to divide and restore the original tissue architecture and function. In order to investigate further the potential application of these three-dimensional scaffolds in tissue engineering, this study was conducted with the aim of using non-woven scaffolds for the *in vitro* proliferation of human and rabbit bone-marrow-derived mesenchymal progenitors. Then, an experimental model of osteochondral lesions in the rabbit knee was used to ascertain the tolerability and safety of these HYAFF11^®^ scaffolds used with or without progenitor cells. In light of the encouraging results obtained, the authors concluded that HYAFF11^®^ non woven meshes are suitable support for tissue engineering, with mesenchymal progenitor cells actively dividing and giving rise to a loose extracellular matrix inside the interfibrillar space of the biomaterial. These pluripotent cells express collagen type IIA, a typical marker of a prechondrogenic state. When this “cellularized construct” was implanted into a rabbit knee cartilage defect, with or without cells, no inflammatory reaction was noted and the scaffolds were almost completely resorbed four months after implantation. Furthermore, in the end of the treatment, inside the HYAFF11^®^ material bone marrow progenitor cells were able to differentiate to chondrocytes which, in turn, at the level of the superficial cartilage layer, integrated with the pre-existing cartilage margins, retaining their morphology. Inside the subchondral bone, the typical modifications of endochondral ossification occurred, such as hypertrophy, calcification, and substitution by cancellous bone.

The same results were obtained by Grigolo *et al*. [45], who used MSC for the treatment of osteoarthritis (OA). With the aim to explore a new approach to treat OA patients suffering from early degenerative lesions of hyaline cartilage, they transplanted in an experimental animal model of OA a hyaluronan-based scaffold (Hyaff11^®^) seeded with Mesenchymal Stem Cells obtained from bone marrow and expanded in culture. Results obtained confirm, with statistically significant differences, the higher quality of the regenerated tissue obtained with scaffolds carrying mesenchymal stem cells compared to the scaffold alone. As a results, the authors concluded, it is possible to demonstrate that HYAFF11^®^, a hyaluronan-based scaffold, provides promise for mesenchymal stem cell implantation and that may have application for the treatment of early OA in humans.

The behavior of MSC embedded in biomaterials, in the long term and in the context of pathological joint, is under extensive characterization. As regards the *in vitro* chondrogenic differentiation of MSCs it requires a complex interaction of growth factors and cell-cell, cell-matrix interactions and the three-dimensional support to maintain their differentiated phenotype *in vitro*. Different factors, like bone morphogenetic proteins (BMPs), insulin growth factor-1 (IGF-1) and transforming growth factor-β (TGFβ) promote chondrogenic effects *in vitro* or *in vivo*. [46]. *In vitro* and *in vivo* data indicate that the differentiation of isolated chondrocytes onto this scaffold allows the progressive increase for known constituents of cartilage matrix and the repair of damaged cartilage. The repair of human cartilage is mainly performed by clinicians using mature chondrocytes grown onto a scaffold. MSCs are a good alternative to chondrocytes for cartilage regeneration. To obtain new information on the sequence of cellular and molecular events during chondrogenesis, Lisignoli *et al.* [47] analysed human MSC chondrogenic differentiation *in vitro* onto a hyaluronic acid biodegradable three-dimensional scaffold, in a defined medium in the presence of two different concentrations of TGFβ1 as chondrogenic growth factor. Cellular proliferation and the expression of chondrogenic genes were analyzed by real-time PCR and immunohistochemical techniques while cell morphological evaluation was performed by light and electron microscopy. With this study the authors demonstrate that chondrogenic differentiation of human MSCs onto a hyaluronic acid based scaffold is dependent on the concentration of TGFβ used. Moreover, they confirm that, since the scaffold is made of hyaluronic acid, as a cellular cross-linker, it may represent a first step in bringing the cells into close contact so favoring interactions with other ECM molecules.

## 8. HYAFF^®^ for *in Vivo* Vessel Regeneration

With the evolution of tissue engineering, the choice of materials for vascular scaffolding has shifted from purely synthetic biomaterials to biological polymers, such as extracellular matrix (ECM) proteins (i.e. collagen, fibrin), that could hypothetically more faithfully elicit native cell responses and likewise regenerate damaged tissue [48].

However, the necessity to chemically stabilize such biopolymers and process them to remove immunogenic donor epitopes can compromise their native biological/mechanical characteristics [48]. Thus, recent studies have focused on ECM-based biomaterials, such as proteoglycans and glycosaminoglycans (GAGs), which could be useful as scaffolds for vascular endothelial cell (EC) regeneration, due to their presence in the vascular intima and media and frequent amenability to chemical stabilizion with little biological alteration [48]. One such GAG is hyaluronan (HA), whose content and form in vessels varies Hyaluronic acid cues for functional endothelialization of vascular constructs state of development and health [48,49].

Artery-Bioregeneration Assist Tube (ABAT) were tested in different animal models. ABAT (HYAFF11^®^ tubules, 2 mm diameter, 1 cm length) were grafted in the abdominal aorta of 30 rats as temporary absorbable guides to promote regeneration of vascular structures [50]. These experiments resulted in three novel findings: 1) complete endothelialization of the tube’s luminal surface occurred; 2) sequential regeneration of vascular components led to complete vascular wall regeneration 15 days after surgery; and 3) the biomaterial used created the ideal environment for the delicate regeneration process during the critical initial phases, yet its biodegradability allowed for complete degradation of the construct four months after implantation, at which time, a new artery remained to connect the artery stumps. This study assessed the feasibility to create a completely biodegradable vascular regeneration guide *in vivo*, able to sequentially orchestrate vascular regeneration events needed for very small artery reconstruction.

The ability of ABAT grafts to develop into neovessels of larger size (4 mm) was tested in a porcine model, focusing on extracellular matrix (ECM) remodeling and elastin biosynthesis [51]. ABAT tubes (diameter 4 mm, length 5 cm) were implanted in an end-to-end fashion in the common carotid artery. All the animals survived the observation period without complications. Intimal hyperplasia (initiating at the anastomotic site) and graft thrombosis led to 3 cases of partial or complete occlusion, as demonstrated by histological examination. There were no signs of stenoses or aneurysms in the remaining grafts. After five months, the biomaterial was almost completely degraded and replaced by a neoartery segment composed of mature smooth muscle cells, collagen and elastin fibers organized in layers furthermore, it was completely covered on the luminal surface by endothelial cells. Whereas in previous small animal studies, patency rates were not optimal, those obtained in the present study using hyaluronan-based grafts of larger size confirmed the ability of these constructs to guide the development of a well-functioning neoartery, with the remarkable additional attribute of facilitating the formation of organized layers of elastin fibers.

## 9. Conclusions

Hyaluronan is a ubiquitous molecule present in all the connective tissues and it has been shown to exert a fundamental role in many biological processes such as water balance, cell recognition, embryonic development and wound healing both in adult and fetal life stages. When considering all these biological properties, for many researchers it has been tempting to think that hyaluronan derivatives may constitute suitable supports for the growth of mammalian cells.

Recently, hyaluronan, a non-sulfated glycosaminoglycan, has been modified by the esterification of the carboxyl groups along the backbone with aromatic alcohols to form a water insoluble biomaterial which can be processed to produce spun fibers, woven and non-woven textiles, meshes, membranes, etc. In this review we showed that the benzyl ester of hyaluronan (HYAFF11^®^ materials) is a very suitable material both for tissue engineering or regenerative medicine. Indeed two commercial cellularized products find large clinical applications for skin and for cartilage regeneration. In this latter years, in which large attention is put on stem cells word, the ability of HYAFF11® to influence stem cell behavior open new intrigue applications.
